# Targeting p-FGFR1^Y654^ Enhances CD8+ T Cells Infiltration and Overcomes Immunotherapy Resistance in Esophageal Squamous Cell Carcinoma by Regulating the CXCL8–CXCR2 Axis

**DOI:** 10.3390/biomedicines13071667

**Published:** 2025-07-08

**Authors:** Hong Luo, Liwei Wang, Hui Gao, Daijun Zhou, Yu Qiu, Lijia Yang, Jing Li, Dan Du, Xiaoli Huang, Yu Zhao, Zhongchun Qi, Yue Zhang, Xuemei Huang, Lihan Sun, Tao Xu, Dong Li

**Affiliations:** 1 Department of Oncology, General Hospital of Western Theater Command, Chengdu 610083, China; luohong3062007@sina.com (H.L.); bb235064@139.com (L.W.); gaoh_rad@foxmail.com (H.G.);; 2 Unit 32357 of People’s Liberation Army, Chengdu 611500, China

**Keywords:** p-FGFR1^Y654^, CD8+ T cells, ESCC, CXCL8–CXCR2, MDSCs

## Abstract

**Background:** Esophageal squamous cell carcinoma (ESCC) is a fatal malignant tumor. Several studies have demonstrated that immune checkpoint inhibitors can provide clinical benefits to patients with ESCC. However, the single-agent efficacy of these agents remains limited. Although combination therapies (e.g., radiotherapy, chemotherapy) can help to overcome immunotherapy resistance in ESCC, their severe side effects limit clinical application. This study aimed to explore new resistance mechanisms to immunotherapy in ESCC and identify novel molecular targets to overcome immunotherapy resistance. **Methods:** We employed immunohistochemistry staining to examine the p-FGFR1^Y654^ in tumor samples obtained from 103 patients with ESCC, in addition to evaluating CD8+ T cell infiltration. In vitro expression, western blotting, CCK-8, 5-bromo-2′-deoxyuridine incorporation assays, and migration assays were used to confirm the impact of AZD4547 on p-FGFR1^Y654^ expression and the proliferation and migration in ESCC cell lines. Through RNA sequencing analysis, databases such as the Cancer Genome Atlas (TCGA) and Gene Set Cancer Analysis (GSCA), and the reconstruction of transgenic mice using the humanized immune system, we validated the correlation between the expression of p-FGFR1^Y654^ and CD8+ T cell infiltration. We also explored how p-FGFR1^Y654^ recruits myeloid-derived suppressor cells (MDSCs) through the CXCL8–CXCR2 axis to suppress the therapeutic efficacy of immunotherapy in ESCC. Finally, the tumor-suppressive effects of AZD4547 combined with immunotherapy were confirmed in vivo in tumor-bearing mice with a humanized immune system. **Results:** We found that the inhibition of p-FGFR1^Y654^ expression in ESCC can enhance CD8+ T cell infiltration by suppressing the CXCL8-–XCR2 recruitment of MDSCs. AZD4547, combined with immunotherapy, further promotes immunotherapeutic efficacy in ESCC. **Conclusions:** In conclusion, our study presents a promising model for combination therapy in ESCC immunotherapy.

## 1. Introduction

Esophageal cancer, a common malignancy in China, is increasing annually. It is the sixth-most common malignant tumor worldwide, and esophageal squamous cell carcinoma (ESCC) is especially prevalent in China [1]. Despite advancements in various treatments, the 5-year survival rate remains below 40% [2]. Studies indicate that immune checkpoint inhibitors can benefit ESCC patients. However, the scope of their therapeutic impact is constrained. Current data indicate that the objective remission rate of immune checkpoint inhibitors used alone in the second-line and subsequent treatment of advanced ESCC remains at only about 20% [3,4], and the reasons for this mainly lie in insufficient infiltrating lymphocytes, PD-L1 expression, T cell depletion, tumor-associated macroplyhages, and MDSCs, among other factors, that lead to immunotherapy tolerance [5]. Although resistance can be partially alleviated by combination therapy with other treatments, including radiotherapy and chemotherapy [6,7,8], severe side effects limit its widespread use in clinical practice. Therefore, it is crucial to explore new mechanisms of immunotherapy resistance in ESCC and identify novel molecular targets to enhance efficacy.

Fibroblast growth factors are crucial in various physiological processes by interacting with their receptors, such as angiogenesis, embryogenesis, and trauma repair [9]. FGFR gene has been identified as a key oncogenic driver in a variety of malignancies, sustaining the malignant properties of tumor cells [10]. FGFR amplification, mutation, and fusion are recognized as primary mechanisms that lead to FGFR activation [11], with receptor tyrosine kinase (RTK) phosphorylation acting as a pivotal intermediary in the regulation of tumor cell advancement [12]. FGFR1 autophosphorylation is mediated through a series of precisely ordered intermolecular processes with approximately three main stages. The first stage primarily occurs at the Y653 site, the second stage mainly involves Y853, Y463, Y766, and Y858, and the final stage at Y654, increasing FGFR1’s intrinsic kinase activity 10-fold. Therefore, FGFR1 autophosphorylation at the Y654 site (p-FGFR1^Y654^) is closely related to FGFR1 signaling pathway activation [13].

Immunotherapy has delivered unprecedented clinical advantages in treating lung cancer and other malignancies. However, this benefit may predominantly aid patients with tumors already infiltrated with CD8+ T cells [14]. In contrast, patients with tumors characterized by a low mutation load or lacking specific driver genes (such as ALK/EGFR) may have a reduced therapeutic response to anti-PD-1/PD-L1 interventions. This reduced efficacy is primarily attributed to the absence of CD8+ T cells that recognize tumor neoantigens [15]. One study discovered that FGF2 overexpression in fibroblasts significantly upregulated depleted T cells by the single-cell sequencing of tissue sources from eight patients with ESCC and that killing T cell toxicity could be improved using the FGFR inhibitor AZD4547 [16]. Multiple studies have shown that chemokines can recruit MDSCs to tumor sites. MDSCs, a diverse set of immature precursor cells, are unable to mature into granulocytes, macrophages, or dendritic cells, instead constituting a population of innate cells that suppress the immune response. These cells exert an immunosuppressive effect in the tumor microenvironment, impacting both innate and adaptive immune responses through a range of mechanisms. Notably, one of the key mechanisms includes impeding the infiltration of CD8+ T cells, which can result in a dampened immune response by triggering the programmed cell death of these T cells [17,18]. However, it remains unclear whether the activation of the FGFR signaling pathway caused by FGFR1 auto-phosphorylation can exert an immunosuppressive effect in ESCC by regulating the tumor microenvironment.

In our previous study, we identified p-FGFR1^Y654^ in four human ESCC cell lines, with TE10 exhibiting high and KYSE150 showing low expression, which clarified that p-FGFR1^Y654^ expression could be used as a marker of mesenchymal cell origin in ESCC. This study investigated p-FGFR1^Y654^ expression and the CD8+ T cell infiltration of 103 ESCC samples to explore their association with prognosis. The analysis of transcriptomic data and TCGA and GSCA databases further validated it. Additionally, we conducted experiments on transgenic mice with a humanized immune system to study the relationship between p-FGFR1^Y654^ expression and CD8+ T cell infiltration and the mechanisms underlying this relationship. Our study presents a promising model for combination therapy in ESCC immunotherapy.

## 2. Methods

### 2.1. Patient Information and Public Database Sources

A total of 103 ESCC patients who underwent surgery at the Western Theater Command General Hospital from January 2017 to July 2020 were retrospectively analyzed. The criteria for inclusion were (1) patients who underwent surgical treatment and had a definitive diagnosis of ESCC through pathological histology post-operation; (2) absence of prior antitumor treatment before surgery; (3) availability of comprehensive clinical and pathological information for the patient. The criteria for exclusion included (1) mixed cell carcinoma containing ESCC; (2) combination with other acute lethal diseases; (3) insufficient tissue specimens for complete immunohistochemistry (IHC) testing; (4) combination with other more severe psychiatric and organic diseases. Gender, age, and tumor information (size, location, grade, stage) were collected from all patients through the hospital inpatient system. The cut-off date for patient follow-up was 6 January 2021.

TCGA database downloaded RNA sequencing data and patients’ clinical data, and the database used in this study was published by TCGA-ESCA (*n* = 174). The GSCA database was used for the online analysis of chemokine mutation status.

### 2.2. Immunohistochemistry

Immunohistochemistry (IHC) was carried out as described [19]. Sample sections were stained with p-FGFR1^Y654^ and CD8 antibody. The IHC positive criteria were as follows: p-FGFR1^Y654^ coloring site in the cell membrane, cytoplasm, and nucleus, presenting brownish-yellow or yellowish-brown granules. IHC stained sections were scanned and recorded with an OLYMPUS-VS200 fully automated pathology slide scanner (OLYMPUS, Tokyo, Japan), and the scanned images were analyzed using the Aipathwell software (Aipathwell v2) for image analysis. H-SCORE was recorded. No attrition or dropout of subjects was observed throughout the entire study period. Patient information including age and gender is displayed in Table 1. Subjects were allocated to groups based on the H-SCORE (H-SCORE = Σ (pi × i) = (percentage of weakly positive cells × 1) + (percentage of moderately positive cells × 2) + (percentage of strongly positive cells × 3), where pi represents the percentage of positive cells, and i denotes staining intensity (scale: 1 = weak, 2 = moderate, 3 = strong). The grouping method did not involve approaches such as randomization or blinding. All data information is presented in this article.

### 2.3. Cell Culture and Reagents

Human ESCC cell lines TE10 was acquired from CTCC, and KYSE150 was acquired from Shanghai Fu Heng Biotechnology Co. (Shanghai, China). Cells were cultured in RPMI 1640 supplemented with 10% fetal bovine serum (FBS), 1% penicillin/streptomycin, and maintained at 37 °C in a humidified 5% CO_2_ atmosphere. AZD4547 (FGFR inhibitor) was purchased from AstraZeneca (Cambridge, UK) and was made into a 10 mmol/L storage solution with DMSO and freshly prepared to the desired concentration before the experiments. SHR-1210 was purchased from Heng Rui Pharmaceuticals Co. (Lianyungang, China) and injected intravenously at 7.5 mg/kg. It was administered weekly and terminated 21 d after administration.

### 2.4. CCK-8 and BrdU Assay

CCK-8 assay and BrdU assay [20,21] were used for the proliferation test as described. The experimental group was exposed to AZD4547 (500 nM) for 72 h, while the negative control group received an equivalent volume of solvent.

### 2.5. Transwell Migration Assay

Transwell assay was used to test the migration ability of cells as described [22]. The experimental group received 500 nM AZD4547 in the upper chamber, whereas the negative control group was untreated.

### 2.6. Mouse Model

Two mouse models constructed by Vital River Laboratory Animal Technology Co. (Beijing, China) were used in this study, one of which is the NOD.Cg-*B2m^em^*^1^*^Tac^Prkdc^scid^ H2-Ab1^tm^*^1^*^Doi^ Il2rg^tm^*^1^*^Sug^*/JicCrl (NOG-dKO) mouse model, which is based on the NOG mouse to knock down the light-chain genes of MHC-I and MHC-II isoforms B2m and Ab1, which render human peripheral blood mononuclear cells (hu-PBMC) transplantation unable to recognize MHC on the surface of mouse cells for immune attack, thus further extending the experimental window (Figure S1A); the other is the NOD.Cg-*Prkdc^scid^ Il2rg^tm^*^1^*^Sug^* Tg(SV40/HTLV-IL3, CSF2)10-7Jic/JicCrl (NOG-EXL) mouse model, also based on NOG mice, which can achieve the reconstitution of myeloid cells after the transplantation of human hematopoietic stem cells (HSCs; hCD34+) encoding IL-3 and GM-CSF genes, presenting a complete human immune system (Figure S1B).

### 2.7. Flow Cytometry Analysis

The flow cytometry of single cell suspensions from peripheral blood and spleen was performed as described [23].

### 2.8. RNA-Sequencing (RNA-seq) Data

After filtering the raw sequencing data to remove the splice sequences and low-quality reads, DESeq2 analysis was applied to screen for differentially expressed genes (*p* < 0.05). The functional information of the differentially expressed genes was analyzed using cluster analysis, GO and KEGG analysis, and GSEA analysis.

### 2.9. Western Blotting (WB) Analysis

WB analysis was performed as described [24]. Antibodies used in this study are listed in Table S1.

### 2.10. Statistical Analysis

Analytical outcomes were determined utilizing the R statistical package (edition 4.4.0). The presented figures reflect the outcomes of three distinct experimental trials and are depicted as the mean ± SD. Inter-group disparities were scrutinized using the *t*-test or the non-parametric test. Prognosis was assessed by Kaplan–Meier analysis. ROC curves were used to assess the accuracy. The chi-square test was used for categorical variables. *p* < 0.05 indicates a statistically significant difference.

### 2.11. Ethics

The Ethics Committee of the General Hospital within the Western Theater Command sanctioned the utilization of historical patient records (2023EC3-Ky015).

## 3. Results

### 3.1. Expression of p-FGFR1^Y654^ and Infiltration of CD8+ T Cells Correlate with Patient Prognosis

**Figure 1:**
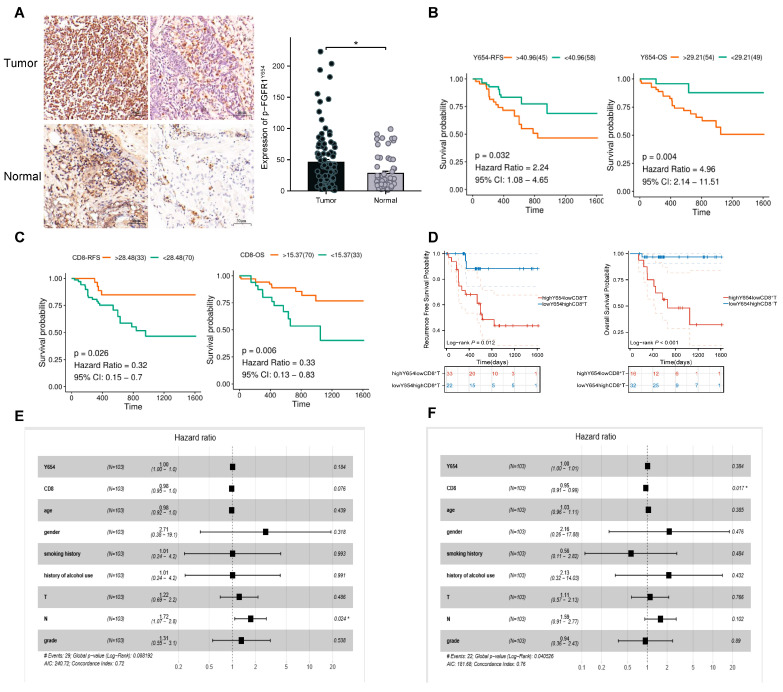
p-FGFR1^Y654^ Expression and CD8+ T cell infiltration are associated with ESCC patients’ prognosis. (**A**) Increased expression of p-FGFR1^Y654^ protein in ESCC tumor tissues compared with adjacent normal tissues (original magnification, ×100; scale bar, 50 µm; mean ± SD; unpaired Student’s *t*-test, * *p* < 0.05). (**B**) Kaplan–Meier curves demonstrating RFS (left) and OS (right) of patients with ESCC according to p-FGFR1^Y654^ expression status. *p*-values are indicated in graphs. (**C**) Kaplan–Meier curves demonstrating RFS (left) and OS (right) of patients with ESCC according to CD8+ T cell infiltration. *p*-values are indicated in the graphs. (**D**) Kaplan–Meier curves demonstrating RFS (left) and OS (right) of patients with ESCC according to p-FGFR1^Y654^ high expression and low CD8+ T cell infiltration (highY654lowCD8+T) and p-FGFR1^Y654^ low expression and high CD8+ T cell infiltration (lowY654highCD8+T). *p*-values are indicated in the graphs. (**E**) Forest plot illustrating the N stage as an independent prognostic factor for RFS by adjusting for clinical characteristics. * The death risk in N+ patients was 1.719 times higher than that in N− patients. (**F**) Forest plot illustrating the CD8+ T cell infiltration as an independent prognostic factor for OS by adjusting for clinical characteristics. * Patients with high CD8+ T cell infiltration had a 0.952-fold increased risk of death compared to those with low infiltration.

The characteristics of the patients are detailed in Table 1 and Table S2. The p-FGFR1^Y654^ expression in tumor and normal tissues was detected using IHC (Figure S2A,B). Tumor tissues showed markedly elevated p-FGFR1^Y654^ levels over normal tissues (* *p* < 0.05; Figure 1A). We also examined CD8+ T cell infiltration in tumor and normal tissues (Figure S2C) and found that tumor tissue did not show any significant changes compared to normal tissue (*p* = 0.11). K–M survival analysis showed that high p-FGFR1^Y654^ expression was associated with poorer RFS and OS (Figure 1B; RFS: *p* = 0.032, 95% CI: 1.08–4.65; OS: *p* = 0.004, 95% CI: 2.14–11.51). However, the CD8+ T cell infiltration was precisely the opposite. Elevated CD8+ T cell infiltration correlated with improved survival outcomes (Figure 1C; RFS: *p* = 0.026, 95% CI: 0.15–0.7; OS: *p* = 0.006, 95% CI: 0.13–0.83). We conducted a joint analysis of the high p-FGFR1^Y654^ expression and low CD8+ T cell infiltration group (highY654lowCD8+T) versus the low p-FGFR1^Y654^ expression and high CD8+ T cell infiltration group (lowY654highCD8+T). We discovered that the latter exhibited a better survival prognosis (Figure 1D; RFS: *p* = 0.012; OS: *p* < 0.001). Univariate Cox regression analysis demonstrated that the N stage was significantly associated with RFS (HR = 1.722, 95% CI: 1.166–2.543). Multivariate Cox regression analysis indicated that the N stage was an independent prognostic factor for RFS. The death risk in N+ patients was 1.719 times higher than that in N- patients (Table S3, HR = 1.719, 95% CI: 1.072–2.757; Figure 1E). Patients with high CD8+ T-cell infiltration exhibited a reduced risk of death (HR = 0.947, 95% CI: 0.908–0.987), as determined by univariate Cox regression analysis of overall survival (OS). Furthermore, CD8+ T cell infiltration was identified as an independent prognostic factor for OS in multivariate Cox regression analysis. Patients with high CD8+ T cell infiltration had a 0.952-fold increased risk of death compared to those with low infiltration (Table S4, HR = 0.952, 95% CI: 0.914–0.991; Figure 1F).

### 3.2. AZD4547 Inhibits p-FGFR1^Y654^ Expression and ESCC Cell Proliferation and Migration In Vitro

**Figure 2:**
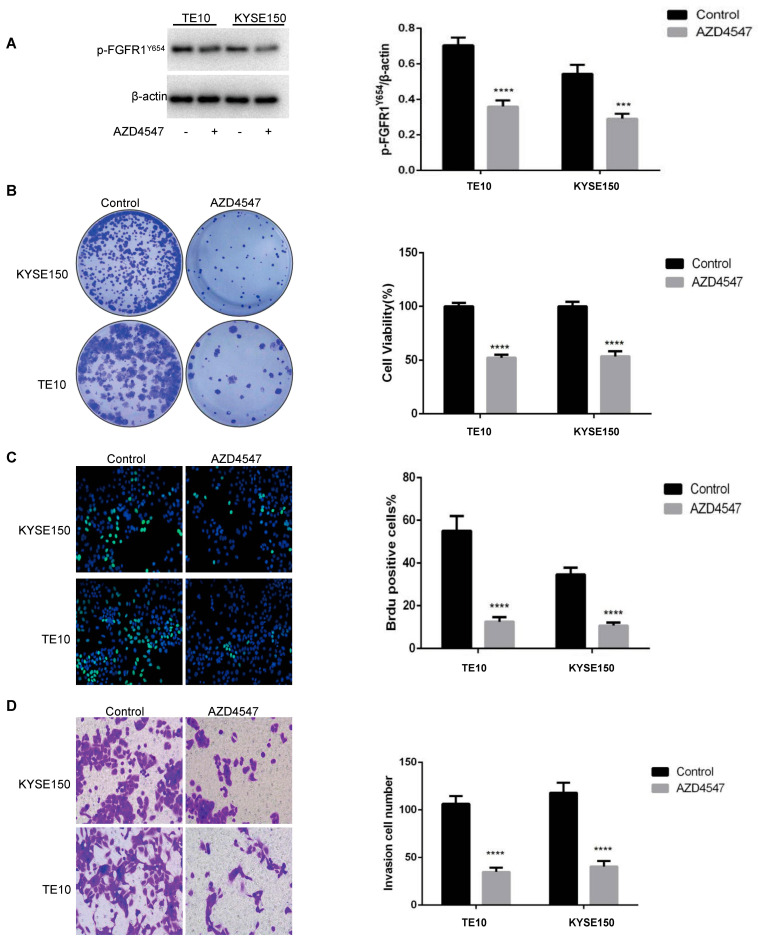
AZD4547 inhibits p-FGFR1^Y654^ expression and tumor cell growth in ESCC. (**A**) The p-FGFR1^Y654^ expression in TE10 and KYSE150 cells after 72 h of AZD4547 (500 nM) treatment detected by WB assay (data are represented as the mean ± SD of three independent experiments; ***, *p* < 0.001; ****, *p* < 0.0001). (**B**) Proliferation of TE10 and KYSE150 cells after 72 h of AZD4547 (500 nM) treatment detected by colony formation assay (data are represented as the mean ± SD of three independent experiments. ****, *p* < 0.0001). (**C**) Proliferation of TE10 and KYSE150 cells after 72 h of AZD4547 (500 nM) treatment detected by BrdU assay (data are represented as the mean ± SD of three independent experiments. ****, *p* < 0.0001). (**D**) Migration of TE10 and KYSE150 cells after 72 h of AZD4547 (500 nM) treatment detected by the Transwell migration assay (data are represented as the mean ± SD of three independent experiments. ****, *p* < 0.0001).

AZD4547, a small-molecule inhibitor of pan-FGFR [25], has exerted antitumor effects in various solid tumors [26,27,28]. A study reported that AZD4547 significantly retarded tumor growth by inducing the mesenchymal–epithelial transition (EMT), reducing ESCC stem cell-like cell populations (CSCs), and inhibiting FGF2-mediated FGFR/ERK signaling [29]. We examined the TE10 cell line with p-FGFR1^Y654^ high expression and the KYSE150 cell line with relatively low expression in ESCC cell lines and observed that AZD4547 inhibited p-FGFR1^Y654^ expression in vitro (Figure 2A). AZD4547’s inhibitory effects on ESCC cell proliferation and migration were confirmed through clone formation, BrdU, and Transwell assays (Figure 2B–D). However, contrary to previous studies [29], we observed that AZD4547 did not inhibit tumor growth alone in vivo when we validated TE10 tumor-bearing hu-PBMC-NOG-dKO mice in vivo (Figure S3A).

### 3.3. Inhibition of p-FGFR1^Y654^ Expression Enhances CD8+ T Cell Infiltration in ESCC

**Figure 3:**
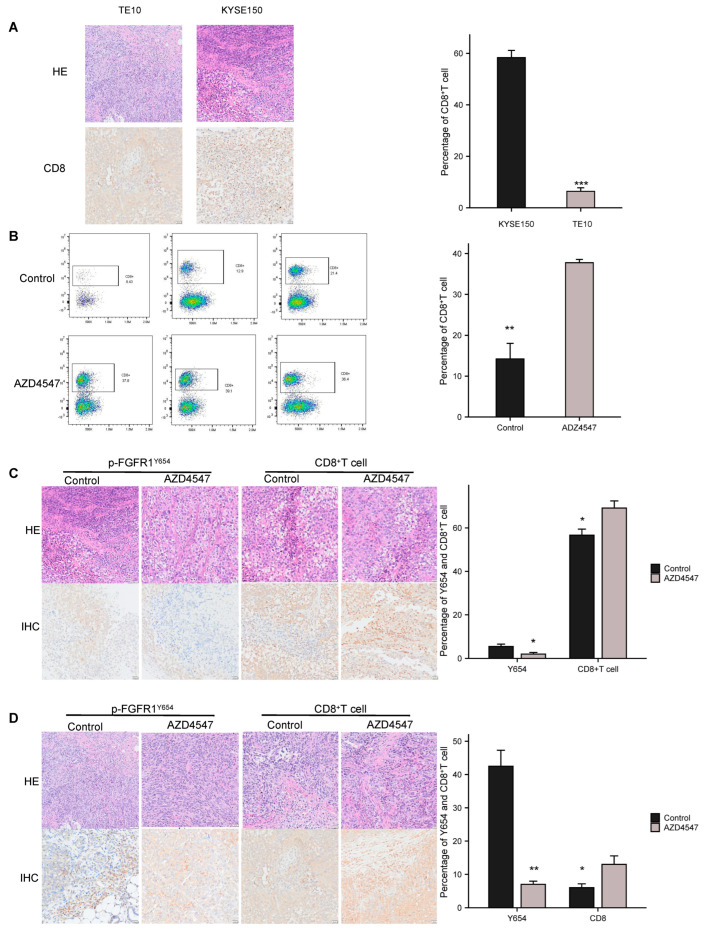
AZD4547 increases CD8+ T cell infiltration in humanized tumor-bearing mice. (**A**) Representative IHC images of CD8^+^ T cells in tumor tissues of TE10 and KYSE150 tumor-bearing hu-PBMC-NOG-dKO mice (original magnification, ×200; scale bar, 50 µm; mean ± SD; *** *p* < 0.001). (**B**) CD8+ T cell infiltration in the spleen of TE10 tumor-bearing hu-PBMC-NOG-dKO mice treated with AZD4547 (10 mg/kg for 14 d), detected by flow cytometric analysis (data are represented as the mean ± SD of three independent experiments, ** *p* < 0.01). (**C**) The p-FGFR1^Y654^ expression and CD8+ T cell infiltration in the tumor tissue of KYSE150 tumor-bearing hu-PBMC-NOG-dKO mice treated with AZD4547 (10 mg/kg for 14 d), detected by IHC staining (original magnification, ×200; scale bar, 50 µm; mean ± SD; * *p* < 0.05). (**D**) The p-FGFR1^Y654^ expression and CD8+ T cell infiltration in the tumor tissue of TE10 tumor-bearing hu-PBMC-NOG-dKO mice treated with AZD4547 (10 mg/kg for 14 d), detected by IHC staining (original magnification, ×200; scale bar, 50 µm; mean ± SD; * *p* < 0.05), ** *p* < 0.01.

To determine if p-FGFR1Y654 is associated with CD8+ T cells in ESCC, we assessed CD8+ T cell infiltration in tissues from TE10 and KYSE150 tumor-bearing hu-PBMC-NOG-dKO mice. We observed significantly decreased CD8+ T cell infiltration in tumors with high p-FGFR1^Y654^ expression (Figure 3A). Flow cytometry analysis revealed that CD8+ T cells in the spleens of TE10 tumor-bearing hu-PBMC-NOG-dKO mice increased significantly following 14 days of AZD4547 treatment at 10 mg/kg (Figure 3B). In the spleens of KYSE150 tumor-bearing hu-PBMC-NOG-dKO mice, no significant changes were observed (Figure S3B). Additionally, the flow cytometry analysis of the peripheral blood from both TE10 and KYSE150 tumor-bearing hu-PBMC-NOG-dKO mice showed no alterations in CD8+ T cell levels (Figure S3C). The IHC analysis of tumor tissues from hu-PBMC-NOG-dKO mice showed that AZD4547 reduced p-FGFR1^Y654^ expression and enhanced CD8+ T cell infiltration (Figure 3C,D). Our findings indicate that FGFR1 autophosphorylation at Y654 may reduce CD8+ T cell infiltration, an effect that can be partially reversed by the FGFR1 inhibitor AZD4547.

### 3.4. RNA-seq Reveals the Potential Mechanism of p-FGFR1Y654 Inhibition by AZD4547 in ESCC

**Figure 4:**
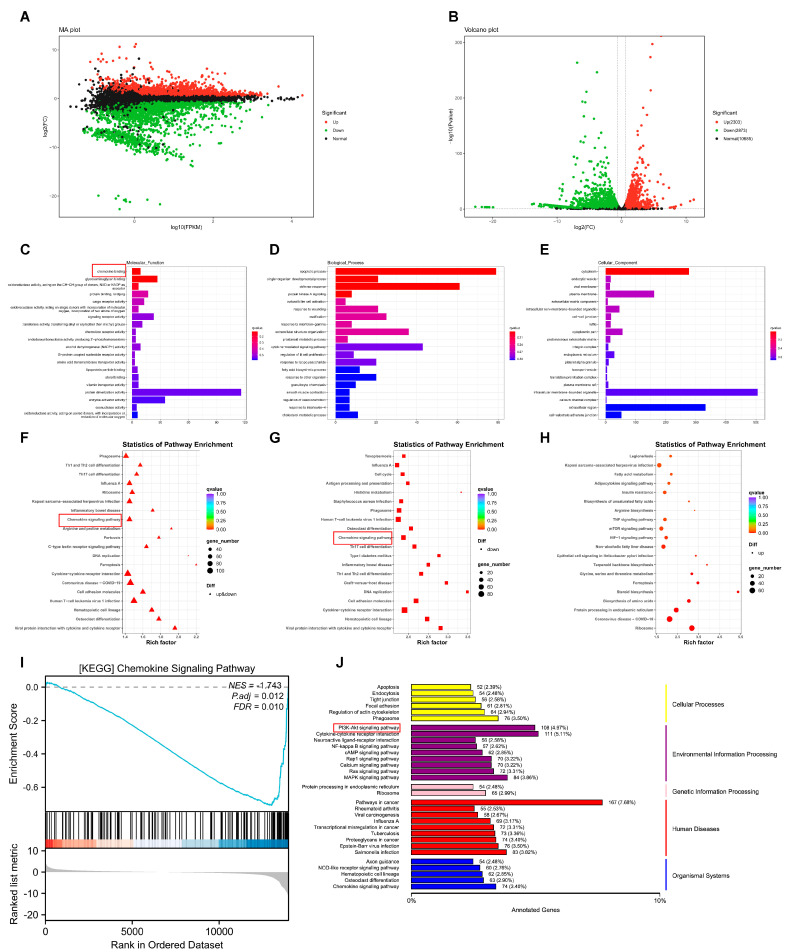
RNA-seq reveals potential molecular mechanisms in ESCC treated with AZD4547. (**A**,**B**). (**A**): Minus-versus-Add (MA) plots indicate gene expression differences between AZD4547-treated TE10 cells and controls. The horizontal coordinate is the A value: log2 (FPKM), that is, the logarithm of the mean expression in the two samples; the ordinate is the M value: |Log_2_(FC)| > 1, that is, the logarithmic value of the multiple of the difference in gene expression between the two samples, which is used to measure the size of the difference in expression. (**B**): Volcano plots indicated folding changes and significance of gene expression differences between AZD4547-treated TE10 cells and controls. The horizontal coordinate represents the logarithm of multiple differences in the expression of a certain gene in the two samples. The ordinate represents the negative value of the statistical significance of the changes in gene expression. Green: Downregulated DEGs (adjusted *p* < 0.05 & Log_2_(FC) < −1), Red: Upregulated DEGs (adjusted *p* < 0.05 & Log_2_(FC) > 1). (**C**–**E**): GO enrichment analysis of the molecular functions, biological processes, and cell components of differentially expressed genes (the ordinate represents the pathway name, the horizontal coordinate represents the number of genes enriched in the pathway, and the column color represents the *p*-value/*q* value). (**F**–**H**): The KEGG pathway enriched all differentially expressed genes, downregulated differentially expressed genes, and upregulated differentially expressed genes (each point represents a KEGG pathway, the ordinate represents the path name, and the horizontal coordinate represents the enrichment factor). (**I**). GSEA indicates evidence for the chemokine signaling pathway is downregulation in KEGG (NES = –1.743; *p*.adj = 0.012, FDR = 0.01). (**J**). The annotation results of differentially expressed gene KEGG classified according to the pathway types in KEGG (the ordinate is the name of the metabolic pathway, and the horizontal coordinate is the number of genes annotated to the pathway and their proportion to the total number of genes annotated).

To understand the potential mechanism by which AZD4547 functions in ESCC, we treated TE10 with AZD4547 (500 nm) for 3 d as a test group and left untreated as a negative control. Of the 5176 differentially expressed genes, 2303 were upregulated and 2873 were downregulated between the two groups (Figure 4A,B). Functional enrichment analysis provided critical insights into the biological pathways modulated by AZD4547. The GO enrichment analysis of all DEGs (Figure 4C–E) showed that both upregulated and downregulated DEGs were significantly associated with the chemokine pathway (Figure S4A–C). These findings align with our hypothesis that p-FGFR1^Y654^ regulates immune evasion via chemokine networks. KEGG pathway analysis further corroborated these results: the analysis of KEGG enrichment for DEGs (Figure 4F–H) indicated an enrichment of the chemokine signaling pathway in all DEGs and downregulated DEGs. GSEA enrichment analysis also confirmed the significant downregulation of genes in the chemokine signaling pathway (Figure 4I). The DEGs’ KEGG annotation categorized by pathway type revealed that genes within the PI3k-AKT signaling pathway were the most abundant and constituted the largest proportion of the annotated genes (Figure 4J). We also verified that AZD4547 inhibits p-AKT expression in TE10 cells. Given that PI3K-AKT activation promotes immunosuppression by upregulating PD-1 [30], this pathway’s inhibition likely synergizes with chemokine axis disruption to enhance T-cell infiltration. Collectively, these data revealed that the inhibition of p-FGFR1^Y654^ expression in ESCC may play a critical role by downregulating the chemokine pathway.

### 3.5. p-FGFR1^Y654^ Recruits MDSCs in ESCC Through the CXCL8–CXCR2 Signaling Axis

**Figure 5:**
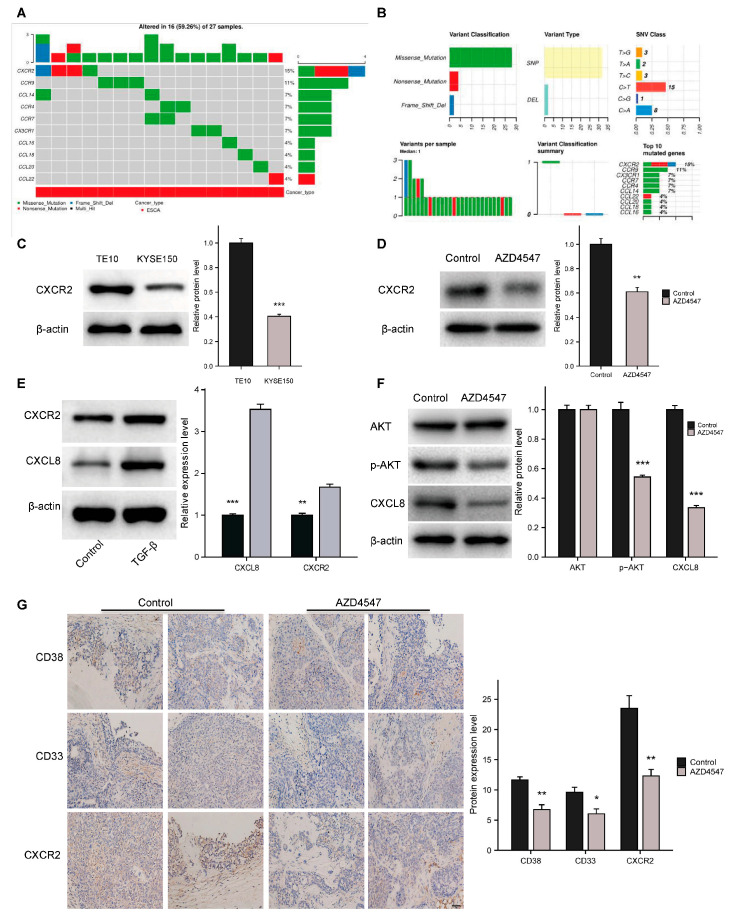
AZD4547 inhibits MDSC recruitment through CXCL8–CXCR2 in ESCC. (**A**,**B**). Mutation profile analysis of chemokine and chemokine receptors by using GSCA datasets. (**C**,**D**). CXCR2 expression measured by western blotting in TE10 and KYSE150 cells and in TE10 cells treated with AZD4547 (500 nM) for 3 d (the data are represented as mean ± SD of three independent experiments, *** *p* < 0.001, ** *p* < 0.01). (**E**). Expression of CXCL8 and CXCR2 in KYSE150 cells treated with TGF-β (5 ng/mL for 72 h) (data are represented as mean ± SD of three independent experiments, *** *p* < 0.001, ** *p* < 0.01). (**F**). Expression of AKT, p-AKT, and CXCL8 in TE10 cells treated with AZD4547 (500 nM) for three days (data are represented as the mean ± SD of three independent experiments, *** *p* < 0.001). (**G**). Representative IHC images of CD38+ cells, CD33+ cells, and CXCR2+ cells in the TE10 tumor-bearing hu-HSC-NOG-EXL mice treated with AZD4547 (10 mg/kg for 21d) and control PBS (the data are represented as mean ± SD in six mice; original magnification, ×200; scale bar, 50 µm; mean ± SD; * *p* < 0.05, ** *p* < 0.01).

To further clarify the critical role of chemokines in suppressing p-FGFR1^Y654^expression, we analyzed the mutations of all chemokines in esophageal cancers using the GSCA database mutation module and found that CXCR2 exhibited the highest mutation rate (Figure 5A,B). Studies have reported that the mRNA expression of CXCL8, a ligand for CXCR2, is markedly higher in tumor tissues, which is expected to be a candidate biomarker for ESCC [31]. Using the TCGA database, we confirmed that both CXCR2 and CXCL8 are overexpressed in ESCC (Figure S5A), whereas CXCL8 exhibited a high diagnostic value for esophageal cancer (Figure S5B; AUC: 0.95, 95% CI: 0.893–1.000). Further analysis using the K–M survival curve revealed that elevated levels of CXCL8 in esophageal carcinoma were linked to unfavorable outcomes (Figure S5C). A study reported that macrophage-derived CXCR2 might be crucial in promoting FGFR signaling-regulated breast tumor formation. CircFGFR1 upregulates CXCR4 expression in gliomas through hsa-miR-224-5p, which also plays a crucial role in tumor growth [32,33]. We detected that CXCR2 expression in TE10 cells was higher than in KYSE150 cells. AZD4547 treatment significantly reduced CXCR2 expression in TE10 cells (Figure 5C,D) and decreased the levels of CXCL8 and p-AKT (Figure 5E). TGF-β is crucial for inducing the metastatic ability of tumor cells by promoting EMT. As TGF-β levels rise, EMT leads to epithelial tumor cells losing adhesion, polarity, and tight junctions by reducing tight junction proteins such as Zonula Occludens-1, E-cadherin, and Occludin, thus acquiring a highly migratory and invasive mesenchymal phenotype [34]. Our study found that CXCL8 and CXCR2 expression significantly increased in KYSE150 cells after TGF-β treatment (Figure 5F).

MDSCs are the most critical immunosuppressive cells in the immune microenvironment, and their recruitment is affected by many factors [17]. In ESCC patients, high MDSC infiltration correlates with a worse prognosis and CD38 serves as a marker for these cells [35]. A recent review indicates that CD33 can be used instead of CD11b to identify human MDSCs, with M-MDSCs showing higher CD33 expression than that in PMN-MDSCs [36]. We also examined the expression of CD38, CD33, and CXCR2 in tumor tissues of TE10 tumor-bearing hu-HSC-NOG-EXL mice treated with AZD4547 10 mg/kg for 21 d and AZD4547 treatment significantly reduced the CD38, CD33, and CXCR2 levels in tumor tissues (Figure 5G). Therefore, our results revealed that p-FGFR1^Y654^ recruits MDSCs in ESCC through the CXCL8–CXCR2 signaling axis.

### 3.6. In Vivo Validation of Tumor Suppression Effect of AZD4547 Combined with SHR-1210

**Figure 6:**
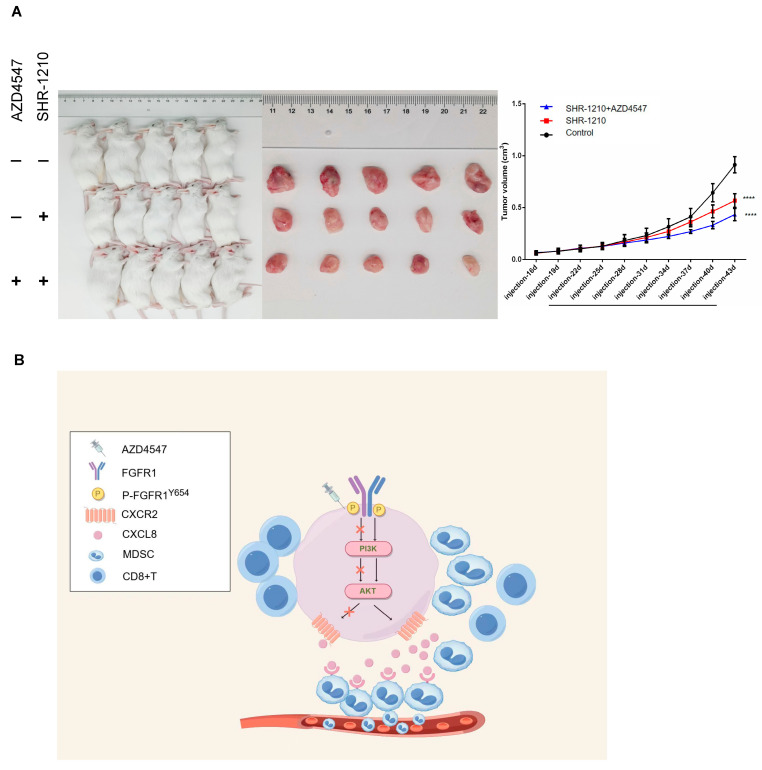
AZD4547 combined with SHR-1210 inhibits tumor growth in vivo. (**A**) AZD4547 combined with SHR-1210 inhibits tumor growth in the TE10 tumor-bearing hu-HSC-NOG-EXL mice (five mice in each group). (**B**) A potential mechanism that inhibits p-FGFR1^Y654^ expression inhibits MDSCs recruitment through CXCL8–CXCR2 in ESCC. ****, *p* < 0.0001.

We administered a combination of AZD4547 and SHR-1210 to TE10 tumor-bearing hu-HSC-NOG-EXL mice and found that the combined therapy markedly suppressed tumor growth more than SHR-1210 alone (Figure 6A).

## 4. Discussion

While FGFR family members have gained importance in solid tumors [37], their potential in immunotherapy remains underappreciated, impeding cancer treatment progress. In ESCC, FGFR1 amplification is more prevalent than esophageal adenocarcinoma, and an overexpression of the FGFR1 gene is shown to independently predict prognosis in ESCC patients [38,39]. However, FGFR1 auto-phosphorylation at the Y654 site can promote FGFR1 signaling pathway activation [13]. In ESCC, EMT can be induced by GSK-3, and PD-L1 expression is markedly increased in EMT-transformed tumor cells. They can induce the apoptosis of T cells compared to primitive epithelial-type tumor cells [40]. Therefore, we hypothesized that p-FGFR1^Y654^, a marker of the mesenchymal phenotype in ESCC, may exhibit a specific relationship with T cells. Transforming the immunotherapy-resistant tumor microenvironment characterized by low T cell infiltration into one that actively promotes antitumor T cell infiltration is an effective strategy. Our study revealed that in ESCC patients, p-FGFR1^Y654^ levels were notably higher in tumor tissues compared to normal tissues and were linked to poor outcomes, while higher CD8+ T cell infiltration indicated better prognosis. Despite the lack of a significant negative correlation between the expression of these two genes in the entire research population, a good prognosis was observed in the lowY654highCD8+T group by combined analysis.

To elucidate the in vivo link between p-FGFR1^Y654^ expression and CD8+ T cells, humanized immune system reconstructed mice were used. Many studies on tumor immunity in humans often rely on mouse models for inference. However, significant disparities exist between human and murine T and B cell signaling, Th1/Th2 differentiation, toll-like receptors, cytokines, and their receptors [41]. To address this issue, various humanized mouse models have been developed in recent years to study the human immune system. The most commonly used humanized mouse models are *Il2rγ*^null^ mouse strains lacking immune function, including NOG (*NOD/Shi-Prkdc^scid^ Il2rγ^tm^*^1^*^Sug^/Jic*) mice or NSG (*NOD/LtSz-Prkd^cscid^ Il2rγ^tm^*^1^*^Wjl^/J*) mice, which can be transplanted with human tissues, HSCs, or PBMCs to analyze human immune cells. However, severe xeno-graft-versus-host disease in mice renders the test window very short, so a team of researchers developed NOG-dKO mice (NOD.Cg-*Prkdc^scid^ Il2rg^tm^*^1^*^Sug^ H2-Ab1^tm^*^1^*^Doi^ B2m^tm^*^1^*^Unc^/Jic* (*NOG-Iab^null^ B2m^null^: N O G–dKO*)) that lack MHC classes I and II [42].

Two mouse models constructed by Vital River Laboratory Animal Technology Co. were employed in this study: the NOG-dKO (NOD.Cg-*B2m^em^*^1^*^Tac^Prkdc^scid^ H2-Ab1^tm^*^1^*^Doi^ Il2rg^tm^*^1^*^Sug^*/JicCrl) mouse model and NOG-EXL (NOD.Cg-*Prkdc^scid^ Il2rg^tm^*^1^*^Sug^* Tg (SV40/HTLV-IL3, CSF2)10-7Jic/JicCrl) mouse model. The former was used to construct hu-PBMC-NOG-dKO by injecting human PBMC for the assessment of CD8+ T cell infiltration, and the latter was used to construct hu-HSC-NOG-EXL by injecting human umbilical cord blood-derived HSCs for the assessment of MDSCs. In vivo experiments verified that inhibiting p-FGFR1^Y654^ expression promoted CD8+ T cell infiltration.

To explore the potential mechanism of inhibiting p-FGFR1^Y654^ expression to promote CD8+ T cell infiltration, we observed that AZD4547-treated TE10 cells were mainly enriched in the chemokine pathway by transcriptome analysis, and CXCL8–CXCR2 played a crucial role. Chemokines can recruit blood-borne cells into tissues, have chemotactic effects on most non-blood-borne cells, and can also be used as biomarkers and targets for cancer therapy [43]. Interestingly, CXCL12/CXCR4 has also been reported in ESCC, and the CXCR4 expression level in primary and metastatic foci was significantly higher than that in normal tissues, and the CXCR4 mediated lymphatic metastasis and distant organ metastasis of ESCC. In addition to metastasis, the CXCL12/CXCR4 signaling pathway contributes to the growth and proliferation of ESCC and is associated with patient prognosis [44,45]. Chemokines and their receptors are highly promiscuous (multiple chemokines can bind to one receptor) and multiplexed (one chemokine can bind to multiple chemokine receptors). This redundancy of chemokines is one of the challenges in targeted chemokine therapy [43,46]. A study reported that the expression of CXCR4 and CXCR2 is positively correlated in gastric cancer and that a positive feedback loop is formed between P65/CXCR4 and STAT3/CXCR2, which induces EMT and promotes metastasis. By establishing CXCR4 and CXCR2 double-knockout cell lines, it was observed that the simultaneous inhibition of CXCR4 and CXCR2 exhibited higher antitumor effects than the inhibition of CXCR4 or CXCR2 alone in vitro and in vivo, thus supporting the theoretical basis of CXCR4/CXCR2 co-suppression therapy [47]. Therefore, whether this crosstalk between chemokines also exists in ESCC should be further explored in subsequent studies.

Comparatively, the relationship between chemokines and MDSCs has been more precisely elucidated in terms of their function in the tumor-immune microenvironment. M-MDSCs, along with monocytes, are predominantly drawn to the tumor by chemokines secreted by the tumor cells, including CCL2, CCL5, and CSF1. The directional movement of PMN-MDSCs is modulated by the release of C-X-C motif chemokines from tumor cells, including CXCL1, CXCL5, CXCL6, CXCL8, and CXCL12 [48]. Some studies have indicated that tumor-secreted CCL20 activates granulocyte–monocyte progenitor cell differentiation through its receptor CCR6, leading to the expansion of PMN-MDSCs, which activates the CXCR2/NOTCH1/HEY1 signaling pathway to increase ALDH+ breast cancer tumor stem cells [49]. The CXCR2 antagonist SB225002 reduced PMN-MDSC aggregation and increased CD8+ T cell infiltration in gastric cancer, further enhancing the antitumor effect of anti-PD-1 [50]. In this study, we characterized the MDSCs of ESCC with CD38 and CD33, verified that AZD4547 could inhibit the recruitment of MDSCs through the CXCL8–CXCR2 axis in tumor tissues of hu-HSC-NOG-EXL mice, and verified the tumor suppressive effect of AZD4547 combined immunotherapy in ESCC.

This study has certain limitations. First, the small clinical sample size limits our ability to visually analyze whether p-FGFR1^Y654^ is inversely related to CD8+ T cell infiltration. Therefore, the sample size should be expanded for the sake of clarity. Second, MDSCs of ESCC, characterized by CD38 and CD33, do not distinguish between M-MDSCs and PMN-MDSCs, and we can investigate alternative molecular markers for further clarification. Third, while our data suggest that MDSC modulation occurs via CXCL8–CXCR2 axis disruption, we acknowledge that this mechanism requires direct functional validation using genetic approaches (e.g., CXCR2 knockout). Recently, scRNA-seq has become a booming technology in understanding the heterogeneity of TME in ESCC [51]. We plan to investigate whether p-FGFR1^Y654^ modulates other chemokines or immune cell populations (e.g., Tregs, NK cells) contributing to the immunosuppressive microenvironment, utilizing techniques like single-cell RNA sequencing and multiplex immunohistochemistry in future studies. At the same time, we will focus on translating these findings into potential clinical applications. We propose a preclinical testing of combined FGFR1 inhibition (specifically targeting p-FGFR1^Y654^) with anti-PD-1/PD-L1 therapy in additional patient-derived xenograft models and syngeneic mouse models to assess long-term efficacy and safety. This data will be crucial for designing future early-phase clinical trials, evaluating this combination strategy in ESCC patients.

In conclusion, this study verified the relationship between FGFR1 auto-phosphorylation at the Y654 site and CD8+ T cells in ESCC by humanized immune system reconstruction. Moreover, the inhibition of p-FGFR1^Y654^ expression can inhibit MDSC recruitment through CXCL8–CXCR2, thereby enhancing CD8+ T cell infiltration (Figure 6B). While these preclinical data identify a valuable combination strategy for ESCC immunotherapy, subsequent clinical studies are warranted to optimize its therapeutic application, particularly evaluating combination-specific toxicities.

## Figures and Tables

**Table 1:** Relationship between p-FGFR1^Y654^ expression and clinicopathological factors.

Clinicopathological Characteristics	p-FGFR1^Y654^		*p*
	Low Expression (*n* = 51)	High Expression (*n* = 52)	
Age, *n* (%)			0.754
≤60	21 (20.4%)	23 (22.3%)	
>60	30 (29.1%)	29 (28.2%)	
Gender, *n* (%)			0.979
Male	47 (45.6%)	49 (47.6%)	
Female	4 (3.9%)	3 (2.9)	
Smoking history, *n* (%)			0.775
Yes	38 (36.9%)	40 (38.8%)	
No	13 (12.6%)	12 (11.7%)	
History of alcohol, *n* (%)			0.203
Yes	38 (36.9%)	44 (42.7%)	
No	13 (12.6%)	8 (7.8%)	
Tumor size, cm (mean ± sd)	3.880 ± 1.760	3.694 ± 1.801	0.530
T Stage, *n* (%)			0.514
T1	3 (2.9%)	6 (5.8%)	
T2	11 (10.7%)	11 (10.7%)	
T3	32 (31.1%)	33 (32.0%)	
T4	5 (4.9%)	2 (1.9%)	
N Stage, *n* (%)			0.687
N0	29 (28.2%)	29 (28.2%)	
N1	14 (13.6%)	12 (11.6%)	
N2	6 (5.8%)	10 (9.7%)	
N3	2 (1.9%)	1 (1.0%)	
Stage, *n* (%)			0.270
I	4 (3.9%)	10 (9.7%)	
II	24 (23.3%)	20 (19.4%)	
III	22 (21.4%)	22 (21.4%)	
IV	1 (1.0%)	0 (0%)	
Histological grade, *n* (%)			0.165
1	6 (5.8%)	8 (7.8%)	
2	36 (35.0%)	41 (39.8%)	
3	9 (8.7%)	3 (2.9%)	
Tumor site, *n* (%)			0.113
Upper	0 (0%)	2 (1.9%)	
Middle	25 (24.3%)	17 (16.5%)	
Lower	26 (25.2%)	33 (32.0%)	
RFS, *n* (%)			0.056
Recurrence	10 (9.7%)	19 (18.5%)	
No recurrence	41 (39.8%)	33 (32.0%)	
OS, *n* (%)			0.005
Alive	46 (44.6%)	35 (34.0%)	
Dead	5 (4.9%)	17 (16.5%)	

Group comparisons utilized chi-square tests (categorical variables) or *t*-tests (continuous variables). *p*-values < 0.05 indicate statistical significance.

## Data Availability

The datasets used and/or analyzed during the current study are available from the corresponding author on reasonable request.

## Supplementary Materials

The following supporting information can be downloaded at: https://www.mdpi.com/article/10.3390/biomedicines13071667/s1, Supplementary Figure S1. Flowchart of humanized mouse models. (**A**) Human PBMC-transferred NOG-dKO mice inoculated with tumor cells for immuno-oncology studies. (**B**) Human HSC-transferred NOG-EXL mice inoculated with tumor cells for tumor immunotherapy research. Supplementary Figure S2. Representative IHC images of p-FGFR1^Y654^ expression and CD8+ T cell infiltration in ESCC tumor tissues. (**A**) Representative IHC images of expression of p-FGFR1^Y654^ protein in ESCC tumor tissues (original magnification, ×200; scale bar, 50 µm). (**B**) Representative IHC images of expression of p-FGFR1^Y654^ protein in ESCC normal tissues (original magnification, ×200; scale bar, 50 µm). (**C**) Representative IHC images of CD8+ T cell infiltration in ESCC tumors and normal tissues (original magnification, ×200; scale bar, 50 µm; mean ± SD; unpaired Student’s *t*-test, *p* = 0.11); Supplementary Figure S3. AZD4547 alone did not inhibit tumor growth or alter CD8+ T cell infiltration in the peripheral blood of tumor-bearing mice. (**A**) Tumor volume in AZD4547 (10 mg/kg for 14 d) monotherapy TE10 tumor-bearing hu-PBMC-NOG-dKO mice (five mice in each group). (**B**) CD8+ T cell infiltration in the spleen of KYSE150 tumor-bearing hu-PBMC-NOG-dKO mice treated with AZD4547 (10 mg/kg for 14 d), detected using flow cytometric analysis (mean ± SD, Student’s *t*-test, *p* = 0.07). (**C**) CD8+ T cell infiltration in the peripheral blood of TE10 tumor-bearing hu-PBMC-NOG-dKO mice treated with AZD4547 (10 mg/kg for 14 d), detected using flow cytometric analysis (mean ± SD; Student’s *t*-test, *p* = 0.93); Supplementary Figure S4. GO enrichment analysis of downregulated differentially expressed genes. (**A**) GO enrichment analysis of the molecular function in differentially expressed genes (the ordinate represents the pathway name, the horizontal coordinate represents the number of genes enriched into the pathway, and the column color represents *p*-value/*q* value). (**B**) GO enrichment analysis of the biological processes in differentially expressed genes (the ordinate represents the pathway name, the horizontal coordinate represents the number of genes enriched into the pathway, and the column color represents the *p*-value/*q* value). (**C**) GO enrichment analysis of the cell components in differentially expressed genes (the ordinate represents the pathway name, the horizontal coordinate represents the number of genes enriched into the pathway, and the column color represents *p*-value/*q* value); Supplementary Figure S5. CXCL8–CXCR2 is associated with tumorigenesis and poor prognosis of esophageal cancer. (**A**) TCGA dataset analysis of CXCL8 and CXCR2 expression in normal tissues compared to tumor tissues (mean ± SD; Student’s *t*-test, * *p* < 0.05, ** *p* < 0.01). (**B**) Diagnostic value of CXCL8 in esophageal cancer (AUC 0.95, CI: 0.893–1.000). (**C**) The TCGA dataset indicates significantly improved disease-specific survival (Log-rank *p* = 0.034) and OS (Log-rank *p* = 0.011) in CXCl8 low expression in esophageal cancer; Table S1. Antibodies used in this study; Table S2. Relationship between CD8+T cell infiltration and clinicopathological factors; Table S3. Univariate and multivariate Cox regression analyses of RFS; Table S4. Univariate and multivariate Cox regression analyses of OS.

## Abbreviations

AUC	Area Under the Curve
CI	Confidence Interval
CSCs	Cell-Like Cell Populations
DEGs	Differentially Expressed Genes
EMT	Epithelial–Mesenchymal Transition
ESCC	Esophageal Squamous Cell Carcinoma
GO enrichment	Gene Ontology Enrichment
GSCA	Gene Set Cancer Analysis
HR	Hazard Ratio
hu-PBMC	Human Peripheral Blood Mononuclear Cells
IHC	Immunohistochemistry
KEGG	Kyoto Encyclopedia of Genes and Genomes
MDSCs	Myeloid-Derived Suppressor Cells
OS	Overall Survival
p-FGFR1^Y654^	FGFR1 autophosphorylation at the Y654 site
RFS	Relapse-Free Survival
RTK	Receptor Tyrosine Kinase
TCGA	The Cancer Genome Atlas
WB	Western Blotting
