# Supplementary material for: Targeting p-FGFR1Y654 Enhances CD8+ T Cells Infiltration and Overcomes Immunotherapy Resistance in Esophageal Squamous Cell Carcinoma by Regulating the CXCL8–CXCR2 Axis
Source: Biomedicines. 2025 Jul 8;13(7):1667. doi: 10.3390/biomedicines13071667 (PMC12293083; doi:10.3390/biomedicines13071667)
Supplement: Supplementary file 1 [file biomedicines-13-01667-s001.zip › biomedicines-3671099-supplementary.pdf]

biomedicines

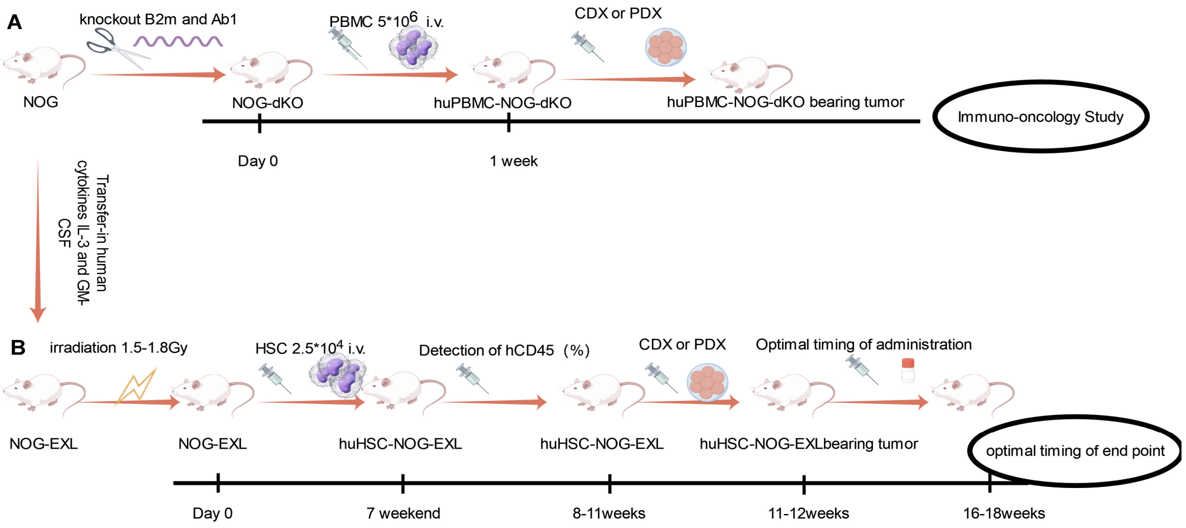

Figure S1. Flowchart of humanized mouse models. (A) Human PBMC-transferred NOG-dKO mice inoculated with tumor cells for immuno-oncology studies. (B) Human HSC-transferred NOG-EXL mice inoculated with tumor cells for tumor immunotherapy research.

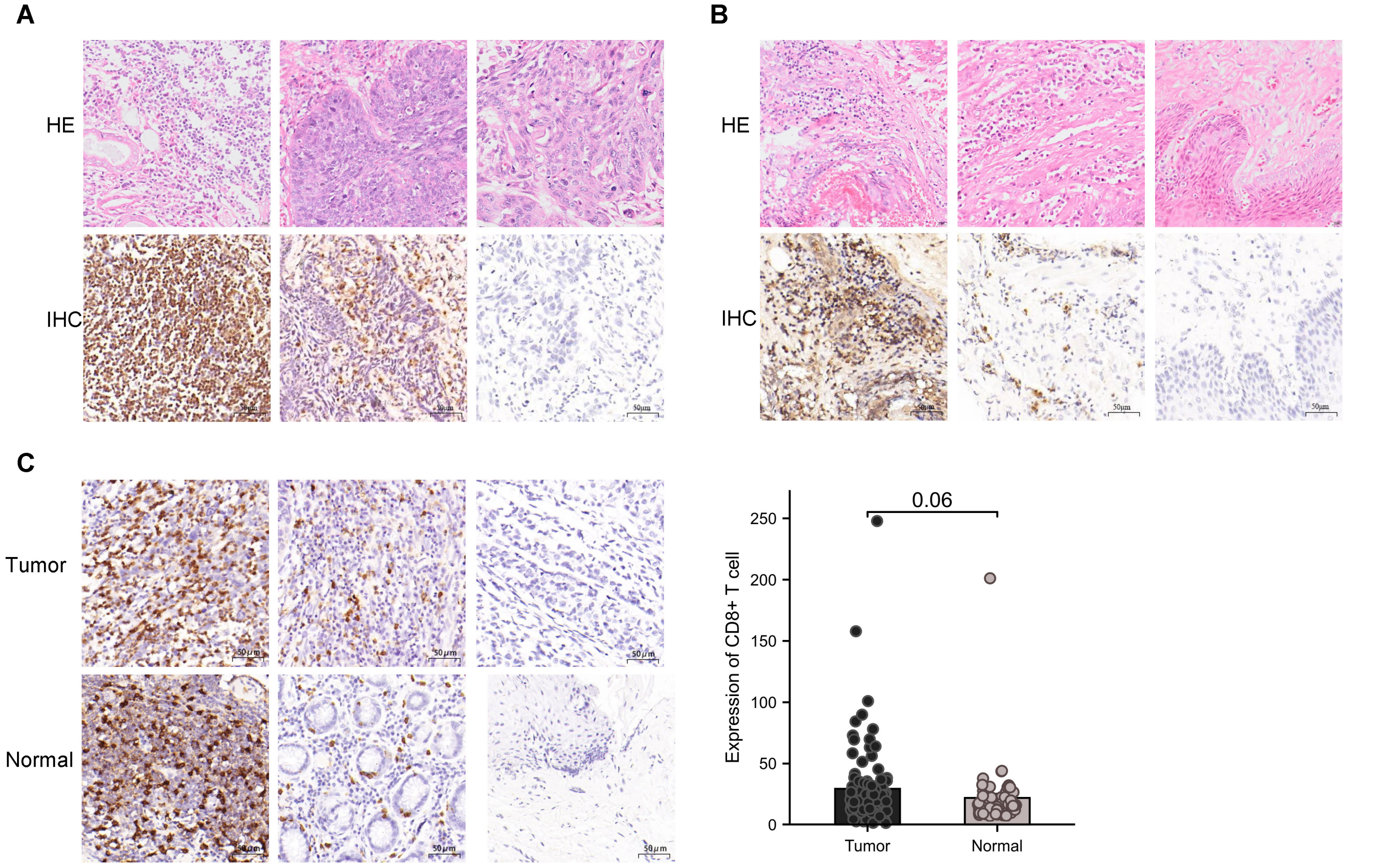

Figure S2. Representative IHC images of p-FGFR1^{Y654} expression and CD8+ T cell infiltration in ESCC tumor tissues. (**A**) Representative IHC images of expression of p-FGFR1^{Y654} protein in ESCC tumor tissues (original magnification, ×200; scale bar, 50 μm). (**B**) Representative IHC images of expression of p-FGFR1^{Y654} protein in ESCC normal tissues (original magnification, ×200; scale bar, 50 μm). (**C**) Representative IHC images of CD8+ T cell infiltration in ESCC tumors and normal tissues (original magnification, ×200; scale bar, 50 μm; mean ± SD; unpaired Student's *t*-test, $p = 0.11$).

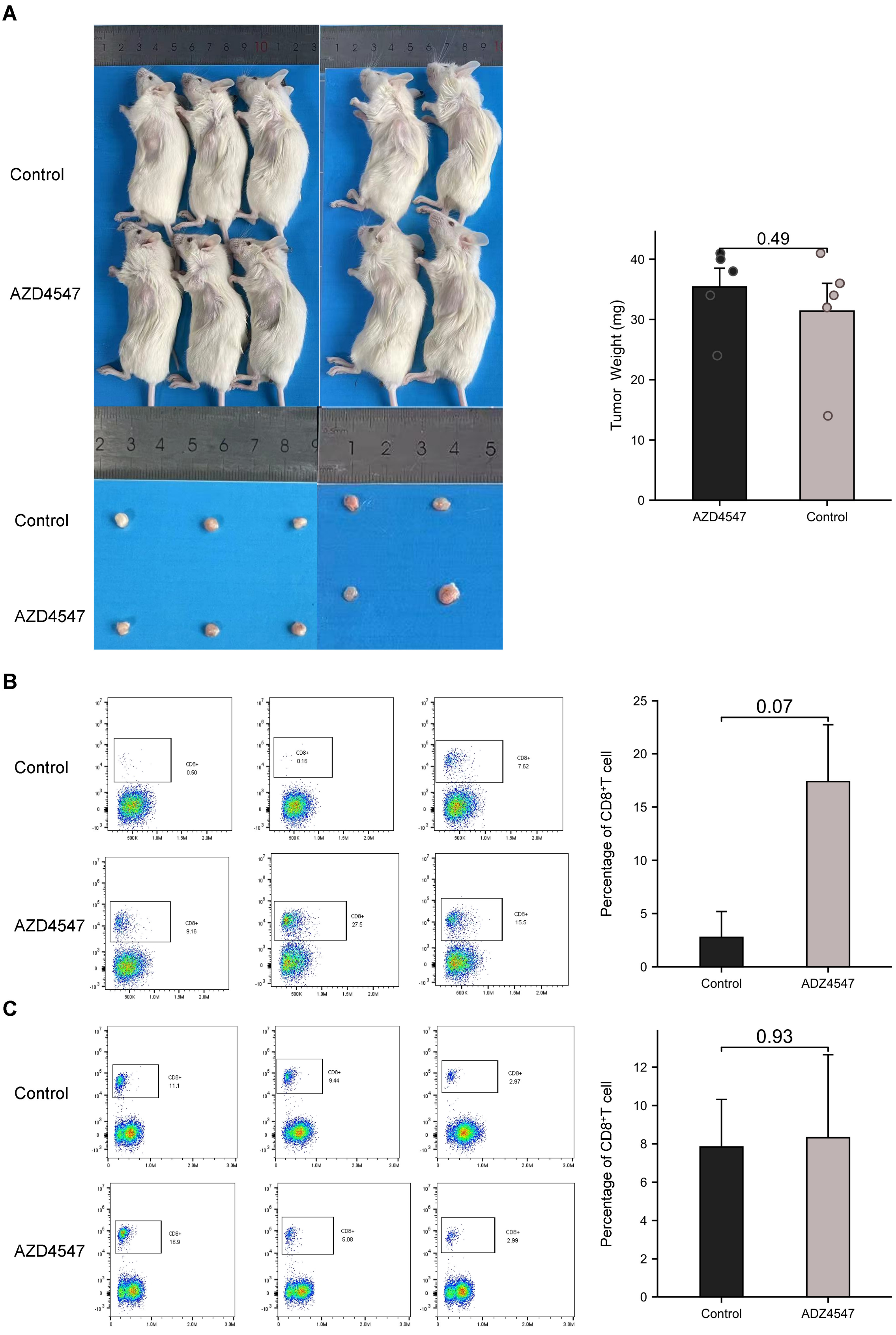

Figure S3. AZD4547 alone did not inhibit tumor growth or alter CD8+ T cell infiltration in the peripheral blood of tumor-bearing mice. (**A**) Tumor volume in AZD4547 (10 mg/kg for 14 d) monotherapy TE10 tumor-bearing hu-PBMC-NOG-dKO mice (five mice in each group). (**B**) CD8+ T cell infiltration in the spleen of KYSE150 tumor-bearing hu-PBMC-NOG-dKO mice treated with AZD4547 (10 mg/kg for 14 d), detected using flow cytometric analysis (mean ± SD, Student's *t*-test,

p = 0.07). (**C**) CD8+ T cell infiltration in the peripheral blood of TE10 tumor-bearing hu-PBMC-NOG-dKO mice treated with AZD4547 (10 mg/kg for 14 d), detected using flow cytometric analysis (mean ± SD; Student's t-test, p = 0.93).

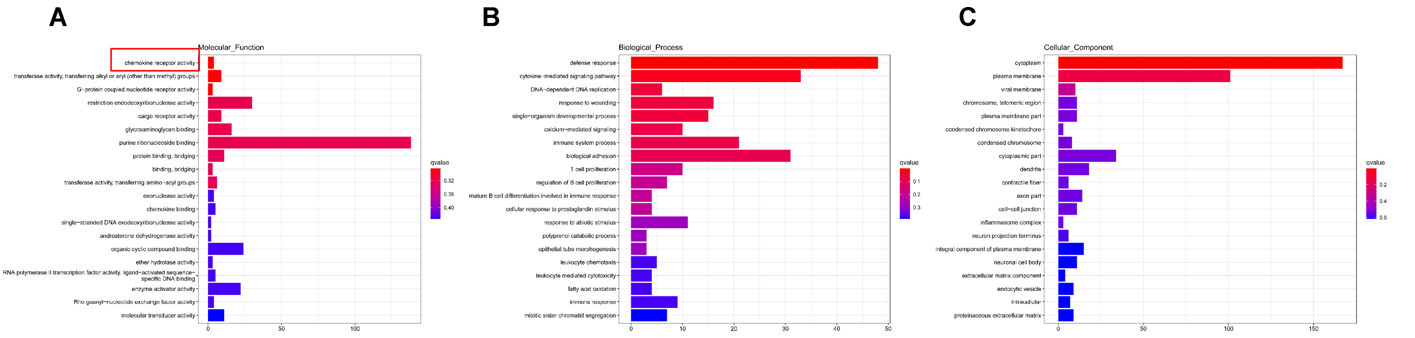

Figure S4. GO enrichment analysis of downregulated differentially expressed genes. (**A**) GO enrichment analysis of the molecular function in differentially expressed genes (the ordinate represents the pathway name, the horizontal coordinate represents the number of genes enriched into the pathway, and the column color represents p-value/q value). (**B**) GO enrichment analysis of the biological processes in differentially expressed genes (the ordinate represents the pathway name, the horizontal coordinate represents the number of genes enriched into the pathway, and the column color represents the p-value/q value). (**C**) GO enrichment analysis of the cell components in differentially expressed genes (the ordinate represents the pathway name, the horizontal coordinate represents the number of genes enriched into the pathway, and the column color represents p-value/q value).

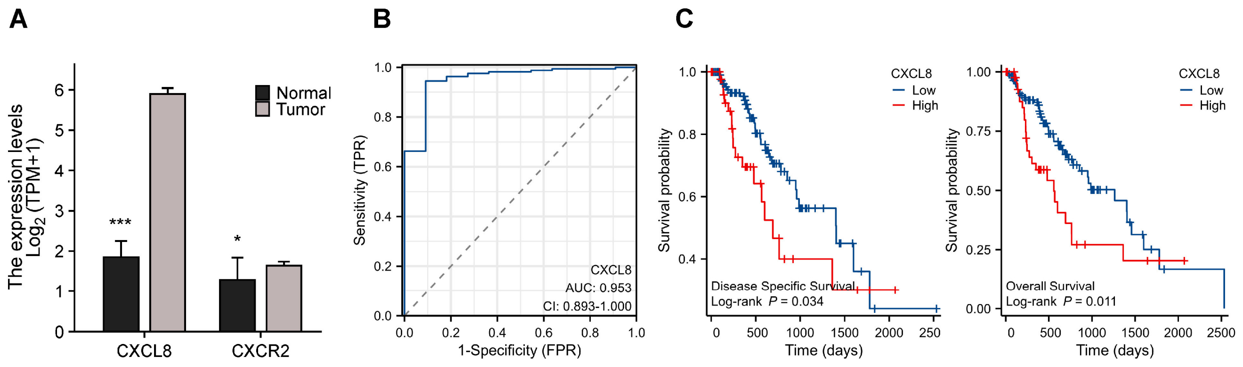

Figure S5. CXCL8–CXCR2 is associated with tumorigenesis and poor prognosis of esophageal cancer. (**A**) TCGA dataset analysis of CXCL8 and CXCR2 expression in normal tissues compared to tumor tissues (mean ± SD; Student's t-test, * $p < 0.05$, ** $p < 0.01$). (**B**) Diagnostic value of CXCL8 in esophageal cancer (AUC 0.95, CI: 0.893–1.000). (**C**) The TCGA dataset indicates significantly improved disease-specific survival (Log-rank p = 0.034) and OS (Log-rank p = 0.011) in CXCl8 low expression in esophageal cancer.

Table S1. antibodies used in this study.

antibodies	source	identifier
CD8	Boster	bs0648R
CD38	Boster	bs0979R
CD33	affnity	DF6789
CXCR2	proteintech	20634-1-AP
CXCL8	Proteintech	27095-1-AP
AKT	Proteintech	10176-2-AP
p-FGFR1Y654	Abcam	ab59194
P-AKT	Abcam	ab38449
TGF-β	MCE	HY-P7118
actin	Serivicebio	GB11001

Table S2. Relationship between CD8+T cell infiltration and clinicopathological factors.

**Clinicopathological characteristics**	CD8+Tcell Low expression （n=51）	CD8+Tcell High expression （n=52）	*P*
**Age，n(%)**			0.932
≤60	22(21.3%)	22(21.3%)	
＞60	29(28.2%)	30(29.1%)	
Gender，n(%)			0.979
Male	47 (45.6%)	49 (47.6%)	
Female	4 (3.9%)	3 (2.9%)	
Smoking history，n(%)			
Yes	36 (35.0%)	42 (40.8%)	0.228
No	15 (14.5%)	10 (9.7%)	
History of alcohol，n(%)			0.433
Yes	39 (37.9%)	43 (41.8%)	
No	12 (11.7%)	9 (8.7%)	
Tumor size，cm(mean ± sd)	3.5 (2.8, 5)	3.5 (2, 4.5)	0.378
T Stage，n(%)			0.178
T1	2 (1.9%)	7 (6.8%)	
T2	13 (12.6%)	9 (8.7%)	
T3	31 (30.2%)	34 (33.0%)	
T4	5 (4.9%)	2 (1.9%)	
N Stage，n(%)			0.370
N0	29 (28.2%)	29 (28.2%)	
N1	14 (13.5%)	12 (11.6%)	
N2	8 (7.8%)	8 (7.8%)	
N3	0 (0%)	3 (2.9%)	
Stage，n(%)			0.458
I	5 (9.8%)	9 (17.3%)	
II	21 (41.2%)	23 (44.2%)	
III	24 (47.0%)	20 (38.5%)	
IV	1 (2%)	0 (0%)	
Histological grade，n(%)			0.370
1	7 (6.8%)	7 (6.8%)	
2	39 (37.8%)	38 (36.9%)	
3	5 (4.9%)	7 (6.8%)	
Tumor site，n(%)			0.153
Upper	1 (2%)	1 (1.9%)	
middle	16 (31.3%)	26 (50.0%)	
Lower	34 (66.7%)	25 (48.1%)	
RFS，n(%)			0.247
Recurrence	17 (16.5%)	12 (11.7%)	
No recurrence	34(33.0%)	40 (38.8%)	
OS，n(%)			0.311
Alive	38 (36.9%)	43 (41.8%)	
Dead	13 (12.6%)	9 (8.7%)	

Table S3. Univariate and multivariate COX regression analyses of RFS.

Factor	Univariate Cox			Multivariate Cox		
	HR	95% CI of HR	*P*	HR	95% CI of HR	*P*
p-FGFR1^{Y654}	1.005	0.998~1.011	0.150	1.004	0.998~1.011	0.184
CD8^{+}T cell	0.977	0.952~1.003	0.080	0.978	0.953~1.002	0.076
Age	1.000	0.950~1.051	0.9p88	0.976	0.918~1.038	0.439
Gender	1.429	0.338~6.038	0.628	2.706	0.384~19.072	0.318
Grade	0.928	0.461~1.868	0.834	1.311	0.554~3.106	0.538
History of smoking	0.894	0.362~2.209	0.808	1.006	0.238~4.245	0.993
History of drinking	0.772	0.314~1.901	0.574	1.008	0.243~4.186	0.991
T	1.550	0.893~2.691	0.119	1.223	0.695~2.151	0.486
N	1.722	1.166~2.543	0.006	1.719	1.072~2.757	0.024

Table S4. Univariate and multivariate COX regression analyses of OS.

Factor	Univariate Cox			Multivariate Cox		
	HR	95% CI of HR	*P*	HR	95% CI of HR	*P*
p-FGFR1^{Y654}	1.005	0.998~1.012	0.158	1.003	0.996~1.011	0.384
CD8^{+}T cell	0.947	0.908~0.987	0.010	0.952	0.914~0.991	0.017
Age	1.040	0.981~1.103	0.187	1.033	0.960~1.112	0.385
Gender	2.039	0.472~8.798	0.340	2.157	0.260~17.884	0.476
Grade	0.911	0.408~2.035	0.820	0.935	0.360~2.426	0.890
History of smoking	0.740	0.269~2.035	0.560	0.561	0.112~2.824	0.484
History of drinking	0.872	0.293~2.595	0.805	2.129	0.323~14.030	0.432
T	1.458	0.790~2.691	0.228	1.105	0.573~2.131	0.766
N	1.529	0.975~2.399	0.064	1.589	0.913~2.765	0.102
